# Function of PIN1 in Cancer Development and Its Inhibitors as Cancer Therapeutics

**DOI:** 10.3389/fcell.2020.00120

**Published:** 2020-03-17

**Authors:** Ji Hoon Yu, Chun Young Im, Sang-Hyun Min

**Affiliations:** New Drug Development Center, Daegu-Gyeongbuk Medical Innovation Foundation (DGMIF), Daegu, South Korea

**Keywords:** cancer therapeutics, PIN1, PIN1 inhibitor, proline-directed phosphorylation, prolyl isomerase, tumorigenesis

## Abstract

Peptidyl-prolyl isomerase (PIN1) specifically binds and isomerizes the phosphorylated serine/threonine–proline (pSer/Thr–Pro) motif, which results in the alteration of protein structure, function, and stability. The altered structure and function of these phosphorylated proteins regulated by PIN1 are closely related to cancer development. PIN1 is highly expressed in human cancers and promotes cancer as well as cancer stem cells by breaking the balance of oncogenes and tumor suppressors. In this review, we discuss the roles of PIN1 in cancer and PIN1-targeted small-molecule compounds.

## Introduction

Proline (Pro)-directed serine/threonine (Ser/Thr) phosphorylation is a modification of various signaling pathways. Proline is the unique amino acid with the ability to have either a cis or trans structure, and these isomerizations are catalyzed by peptidylprolyl isomerases (PPIases). The cis–trans isomerization of proline in the phosphorylated Ser/Thr–Pro motif is mediated by PIN1 (Liou et al., 2011). The PPIase superfamily contains FK506-binding proteins (FKBPs), cyclophilins and parvulins. FKBPs and cyclophilins are inhibited by the immunosuppressants FK506/rapamycin and cyclosporine A (CyA). PIN1 is a kind of parvulins and inhibited by juglone. PIN1 is well known PPIase that controls the isomerization of the phosphorylated Ser/Thr–Pro (pSer/Thr–Pro) motif.

PIN1 contains two domains including an WW domain in N-terminal and a PPIase domain in C-terminal (Lu et al., 1996, 1999). The N-terminal WW domain interacts with specific pSer–Pro or pThr–Pro motifs, which are the regulatory phosphorylation sites of substrate proteins (Lu et al., 1996, 1999). After interacting with its substrate, the PPIase domain isomerizes the pSer/Thr–Pro motifs, which affect the function of protein by the conformational changes of target protein (Lu et al., 1999; Lu P. -J. et al., 2002).

The post-translational modifications of PIN1, containing oxidation, sumoylation, phosphorylation, and ubiquitination, control the PPIase activity and stability of PIN1, and contribute to the high expression and/or activation of Pin1 in cancer development. PIN1 is involved in the cell cycle, synthesized protein folding, and DNA damage responses (Lu et al., 1996). PIN1 is overexpressed in several human cancers (Lee T. H. et al., 2011), including prostate cancer (Ayala et al., 2003; La Montagna et al., 2012), breast cancer (Wulf et al., 2001; Ryo et al., 2002), and oral squamous carcinomas (Miyashita et al., 2003). In cancer patients, a high expression of PIN1 correlates with a poor clinical outcome, lymph node metastasis in non-small cell lung cancer patients, and disease progression in patients with oral squamous carcinoma (Ryo et al., 2001; Ayala et al., 2003; Bao et al., 2004; Suizu et al., 2006). PIN1 overexpression induces chromosome instability and tumorigenesis. PIN1 inactivates and activates more than 26 tumor suppressors and 56 oncogenes, respectively.

In cancer stem cells, multiple PIN1 substrates play an important role. PIN1 regulates the tumorigenesis and expansion of CSCs in leukemia and breast cancer. However, it is not fully understood how PIN1 controls cancer and cancer stem cell development. Several studies have reported that some single nucleotide polymorphisms (SNPs) of the *Pin1* gene increases the cancer risk, whereas other variants function as protective factors (Segat et al., 2007; Lu et al., 2009; Li et al., 2013). In this review, we summarize the function of PIN1 in regulating cancer development and small-molecule compounds that exhibit anticancer activities by targeting PIN1.

## Transcriptional and Post-Translational Regulation of PIN1 in Cancer

Oncogenes activating E2F transcriptional factor including H-Ras, Her2, p38, and PI3K increase the mRNA expression of *Pin1*, which appears to activate *Pin1* transcription by E2F, considering the existence of the E2F consensus sequence in the *Pin1* promoter region (Ryo et al., 2002, 2009; Kamimura et al., 2011). The transcriptional activation of PIN1 is induced by the E2F or by the binding of Notch1 with the *Pin1* promoter region (Ryo et al., 2002; Rustighi et al., 2009). In acute myeloid leukemia (AML), oncogenic CCAAT/enhancer binding protein-α ((C/EBPα)-p30) is a dominant negative isoform of the tumor suppressor C/EBPα that is generated by *CEBPA* mutations. C/EBPα-p30 recruits the E2F transcription factor to bind to the *PIN1* pro-moter.

On the contrary, p53 and AP4 act as transcriptional repressors and reduce the *Pin1* transcription (Mitchell and Smith, 1988; Jeong et al., 2014). Xbp1 induces the transcription of p53 via HEPN1 and represses E2F1 via NF-κB activation, resulting in reduced *Pin1* transcription (Chae et al., 2016). The transcription of PIN1 is repressed by *BRCA1*, a tumor suppressor gene (MacLachlan et al., 2000). BRCA1 interacts with some proteins to control DNA repair. During cancer development, BRCA1 is often mutated, resulting in the accumulation of DNA damage in cells (Mersch et al., 2015). The mRNA stability of *PIN1* is reduced by microRNAs, such as miR-200c (Luo et al., 2014), miR-200b (Zhang et al., 2013) and miR296-5p (Lee et al., 2014) in breast cancer, breast CSCs, and prostate cancer.

Under physiological conditions, the protein activity is generally regulated by post-translational modifications. Post-translational modifications at specific sites, including sumoylation, phosphorylation, ubiquitination, and oxidization, can regulate the PIN1 protein activity and function. The S65, S71, S138, and S16 residues in PIN1 protein sequence are reported as phosphorylation sites (Eckerdt et al., 2005; Rangasamy et al., 2012; Bhaskaran et al., 2013). The PIN1 phosphorylation at Ser16 in the N-terminal WW domain, inhibits the ability of PIN1 to bind with its substrates (Lu P. -J. et al., 2002), and it can be induced by ribosomal S6 kinase 2 (Cho et al., 2012), protein kinase A (Lu K. P. et al., 2002), and aurora kinase A (Lee et al., 2013). The PIN1 phosphorylation at Ser65 in the C-terminal PPIase domain by polo-like kinase (Plk1) (Eckerdt et al., 2005) induces the ubiquitination and stabilization of PIN1. The PIN1 phosphorylation at Ser138 by mixed-lineage kinase 3 induces its nuclear translocation and catalytic activity (Rangasamy et al., 2012). The PIN1 phosphorylation at Ser71 by death-associated protein kinase 1 (DAPK1) can reduce MYC and E2F-mediated oncogenic transformation.

PIN1 sumoylation at Lys6 in the N-terminal WW domain and Lys63 in the C-terminal PPIase domain suppresses its oncogenic function and enzymatic activity (Chen et al., 2013). PIN1 desumoylation at Lys6 and Lys63 by SUMO1/sentrin specific peptidase 1 (SENP1) recovers its substrate-binding and catalytic activity. Under oxidative stress, PIN1 is generally oxidized at Cys113 in the PPIase catalytic site, which can suppress the enzymatic activity of PIN1 (Chen et al., 2015).

PIN1 reduces the degradation of oncogenes and/or growth-promoting regulators, such as β-catenin, AKT, c-fos, cyclin D1, c-Jun, ER, HER2, Hbx, HIF-1, Mcl-1, NF-κB, Nanog, NUR77, PML-RARa, Oct4, Stat3, and Tax (Lu and Zhou, 2007; Gianni et al., 2009; Liao et al., 2009; Moretto-Zita et al., 2010; Lu and Hunter, 2014; Wei et al., 2015). On the contrary, PIN1 induces the degradation of tumor suppressors such as Daxx, FoxO4, Fbw7, GRK2, PML, KLF10, RARa, RUNX3, RBBP8, Smad, SUV39H1, SMRT, and TRF1 (Lu and Zhou, 2007; Lee T. H. etal., 2009; Ryo et al., 2009; de Thé et al., 2012; Lu and Hunter, 2014; Ueberham et al., 2014; Wei et al., 2015). ERα increases the tumor proliferation through regulating the expression of estrogen response element (ERE)-containing genes in breast cancer (Anderson, 2002). PIN1 induces the ERE-binding affinity and transcription activity, and reduces the ERα degradation mediated by E3 ligase E6AP in breast cancer (Rajbhandari et al., 2012, 2014, 2015). Through inhibiting ubiquitination and destabilizing the transcriptional corepressor SMRT, PIN1 increases HER2 activity (Lam et al., 2008; Stanya et al., 2008). PIN1 also increases the activity of NF-κB pathway via inducing the nuclear accumulation of c-Rel, RelA/p65, and v-Rel (Ryo et al., 2003; Fan et al., 2009). Furthermore, it inhibits the p65 ubiquitination mediated by SOCS-1 (Ryo et al., 2003). PIN1 directed NF-κB activation regulates the proliferation of AML, endometrial carcinoma, glioblastoma, and hepatocellular carcinoma (HCC) (Atkinson et al., 2009; Saegusa et al., 2010; Shinoda et al., 2015; Chen et al., 2016). An isoform of p63, ΔNp63 lacking an intact N-terminal transactivational domain is important for cancer development (Murray-Zmijewski et al., 2006). PIN1 reduces the ΔNp63 ubiquitination induced by WWP1 to enhance the proliferation of oral squamous cell carcinoma (Li et al., 2013). PIN1 stabilizes BRD4 protein to increase the migration and proliferation of gastric cancer (Hu et al., 2017). It also upregulates c-Jun, c-Myc, FoxM1, β-catenin, NUR77, and XBP1 (Chen et al., 2012; Helander et al., 2015; Chae et al., 2016; Kruiswijk et al., 2016; Zhu et al., 2016; Csizmok et al., 2018).

## PIN1 and Signal Transduction in Cancer

**FIGURE 1:**
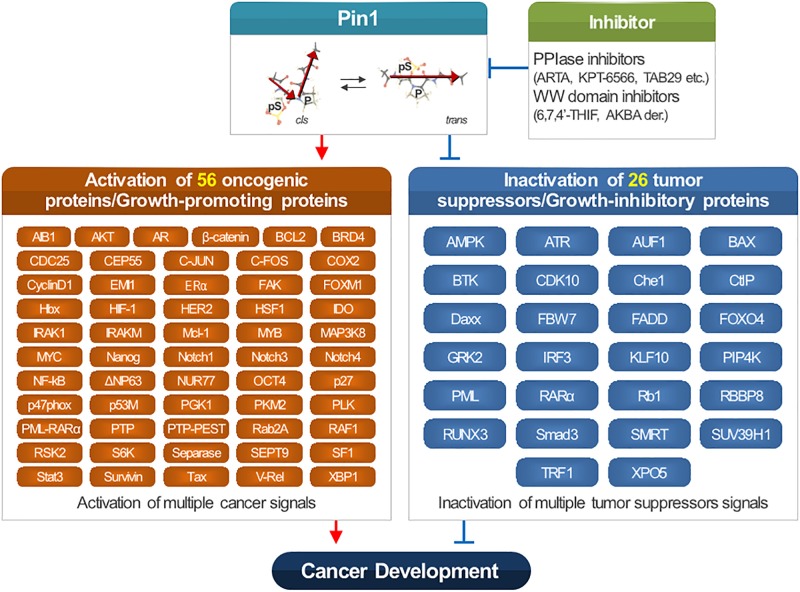
Function of PIN1 in cancer development.

PIN1 is associated with the development of various cancers, including melanoma, breast cancer, gastric cancer, cervical cancer, gallbladder cancer, pancreatic ductal carcinoma, colorectal cancer, prostate cancer, ovarian cancer, non-small cell lung cancer, osteosarcoma, esophageal cancer, hepatitis B virus (HBV)-induced hepatocellular carcinoma, Burkitt lymphoma, and T cell acute lymphoblastic leukemia. PIN1 is reported to activate 56 oncogenes and/or growth-promoting regulators. Also, it is reported to inactivate 26 tumor suppressors and/or growth-inhibitory regulators (Figure 1).

In human breast cancer, PIN1 promotes oncogenesis via the cyclin D1 regulation (Ryo et al., 2001; Wulf et al., 2001). Studies have shown that PIN1 increases cyclin D1 transcription in association with the HER2–HRAS–JNK–AP1, WNT–β-catenin, and NF-κB pathways. PIN1 regulates HER2, NOTCH1, NOTCH3, androgen receptor (AR) and estrogen receptor α (ERα), which are cancer-driving receptors (La Montagna et al., 2012; Rajbhandari et al., 2012). Furthermore, PIN1 regulates AMPK, AKT93, MYC, PKM2, RAF1, SMAD2, SMAD3, STAT3, the RAS family member RAB2A28, FAK, protein tyrosine phosphatase, PTP-PEST, S6K, and SGK1, which act as intracellular signaling modulators (Lee N. Y. et al., 2009; Jo et al., 2015; Chen Y. et al., 2018). PIN1 induces the interaction of non-receptor type 12 (PTP-PEST) with FAK to increase the FAK Tyr397 dephosphorylation, which induces cancer metastasis (Zheng et al., 2009, 2011). PIN1 also promotes epithelial–mesenchymal transition (EMT) of MCF-7 cells by inducing the transcriptional activity of STAT3 and recruiting its transcription coactivator p300 (Lufei et al., 2007). PIN1 induces the cancer metastasis and invasion by activating β-catenin, BRD4, NF-κB, and p53M (Muller et al., 2009; Wang et al., 2012; Zhu et al., 2016; Hu et al., 2017). Overexpression of PIN1 increases the PTOV1 expression as a novel interactome of PIN1, and knockdown of both genes inhibits the expression of β-catenin, cyclin D1, and c-Myc in breast cancer MDA-MB-231 cells (Karna et al., 2019).

Pin1 transgenic mice in mammary glands induces mammary hyperplasia and malignant mammary tumors (Suizu et al., 2006). Pin1-deficient mice inhibit the massive proliferation of breast epithelium in pregnancy through reducing cyclin D1 levels (Liou et al., 2002) and decreases β-catenin expression in breast cancer (Ryo et al., 2001). Pin1 knockout mice show defects in breast development and induces retinal degeneration and neurodegenerative disorder in brain (Fujimori et al., 1999; Liou et al., 2002).

In human liver cancer, PIN1 is associated with the transcription levels of RhoC and RhoA, and co-overexpression of both genes correlates with metastasis and recurrence of HCC (Ng et al., 2019). All-trans retinoic acid (ATRA) is potent PIN1 inhibitor in hepatocellular carcinoma (Liao X. -H. et al., 2017) and co-targeting p53-RS (p53-R249S) with CDK4, c-Myc, or PIN1 is more effective against the treatment of HCC (Liao X. -H. et al., 2017). PIN1 inhibitor (AF-39) significantly suppresses cell proliferation through the XPO5 subcellular distribution and miRNAs biogenesis in HCC cells (Zheng et al., 2019). Inhibition of Pin1 reverses regorafenib resistance in hepatocellular carcinoma (HCC) with reducing EMT, migration and metastasis (Wang et al., 2019).

In pancreatic cancer, PIN1 was highly expressed in pancreatic ductal adenocarcinoma (PDAC) tissues and significantly correlated with the worst outcomes in patients. PIN1 inhibition with specific siRNA or ATRA suppressed tumor growth in PDAC (Chen et al., 2019). Pin1 is overexpressed and correlated with poor prognosis in gastric cancer (Shi et al., 2015). Pin1 inhibition using small molecule inhibitor such as ATRA or short hairpin RNA, reduces cancer development by inhibiting Wnt/β-catenin and PI3K/AKT signaling pathways in gastric cancer (Zhang et al., 2019).

In nasopharyngeal carcinoma (NPC), Pin1 inhibition reduced NPC cell proliferation, colony formation and anchorage-independent growth through the decrease of cyclin D1 expression and the activation of caspase-3 (Xu et al., 2016). Pin1 enhances transcription activity of ATF1 and induces tumorigenesis in NPC (Huang et al., 2016). Using specific siRNA, Pin1-targeted inhibition suppresses transformed properties and prevents cell proliferation in prostate cancer cells (Ryo et al., 2005). In human melanoma metastasis, although the expression of cytoplasmic Pin1 is not associated with primary melanoma clinical outcome, Pin1 expression in cytosol is correlated with poor survival of melanoma patients (Chen X. et al., 2018). In human colorectal cancer, the expression of cytoplasmic Pin1 is importantly correlated with aggressive tumor behaviors and a worse prognosis in colorectal cancer (Pyo et al., 2018).

In osteosarcoma, PIN1 overexpression using adenovirus significantly stimulates MG-63 and U2-OS cell proliferation. Also, PIN1 inhibitor, juglone reduces cell proliferation in osteosarcoma cells (Zhou et al., 2013). In esophageal squamous cell carcinoma (ESCC), increased Pin1 expression is associated with worse outcome of ESCC patients. Also, Pin1 promotes the aggressiveness of ESCC via β-catenin and cyclin D (Lin et al., 2014). In human lung cancer, cancer patients without Pin1 overexpression has longer cancer-related survival than cancer patients with Pin1 overexpression. Pin1 knockdown in H1299 cell reduces cell invasion and migration (Tan et al., 2010).

In metastatic cancer, PIN1 level is considerably higher than that in primary cancer. The TGF-β signaling promotes the metastasis of cancer. PIN1 increases SMAD degradation mediated by E3 ligase Smurf-2 to repress TGF-β signaling (Nakano et al., 2009). In prostate cancer, PIN1 promotes TGF-β-induced metastasis (Matsuura et al., 2010). Inhibiting the phosphorylation of SMAD3 represses the aggressiveness of breast cancer by reducing the interaction with PIN1 (Thomas et al., 2017).

PIN1 is also involved in angiogenesis. It enhances the transcriptional activity and of stability HIF-1α in several cancer cells (Jalouli et al., 2014; Han et al., 2016). PIN1 promotes the VEGF expression mediated by NF-κB in HCC and regulates the transcriptional factors by VEGF including β-catenin and FoxM1 (Wang et al., 2007; Jiang et al., 2015; Shinoda et al., 2015). Overexpression of HIF-1α, VEGF, and *Pin1* is correlated to TAM-resistant MCF-7 cell lines (TAMR-MCF-7) (Oh et al., 2010; Lee T. H. et al., 2011). RNA interference of Pin1 inhibits the angiogenesis as well as the growth of prostate cancer. In TAMR-MCF-7 cells, PI3K/p38 signal pathways increase the *Pin1* expression through increasing E2F1 (Lee K. Y. et al., 2011).

### PIN1 and Signal Transduction in Cancer Stem Cells (CSCs)

Studies have shown a role of PIN1 in stem cells of breast cancer and leukemia (Luo et al., 2014, 2015; Rustighi et al., 2014; Wei et al., 2015). PIN1 induces NOTCH1 cleavage by γ-secretase, leading to enhanced NOTCH1 transcriptional and tumorigenic activities. PIN1 increases NOTCH1 stability to promote self-renewal and metastasis of breast CSCs by reducing the ubiquitin ligase F-box/WD repeat-containing protein 7 (FBXW7)-mediated degradation of NOTCH1 and NOTCH4 (Rustighi et al., 2014). The deletion of *Pin1* decreases the NOTCH-induced invasion of T cell acute lymphoblastic leukemia (T-ALL) cells (Franciosa et al., 2016). PIN1 interacts with the AP1 transcription factors JUN and FOS to activate AP1-dependent RAB2A transcription to promote the expansion and tumorigenesis of breast CSCs (Luo et al., 2015). Overexpression of PIN1 converts normal human breast epithelial cells to cells with stem-like and EMT phenotypes, whereas PIN1 silencing reduces the tumorigenesis and self-renewal activity of breast CSCs in primary breast cancer tissue (Luo et al., 2014, 2015; Rustighi et al., 2014). PIN1 is a pivotal target of miR-200c, a key negative regulator of CSC function and EMT (Shimono et al., 2009; Luo et al., 2014). Inhibition of PIN1 induces the degradation of the fusion oncogene promyelocytic leukemia (PML)–retinoic acid receptor-α (PML–RARα) that drives leukemia stem cells (LSCs), and thereby, treats APL without inducing myeloid differentiation (Ito et al., 2008; de Thé and Chen, 2010). PIN1 controls the maintenance of stability of Nanog, octamer-binding protein 4 (OCT4), and MYC (Nishi et al., 2011; Farrell et al., 2013) and is important for the self-renewal of CSCs.

### Pin1 Regulates the Cell Death Resistance and Inflammation of Cancer

Pin1 inhibits apoptosis through BAX as proapoptotic factor in human eosinophils (Shen et al., 2009) and regulates death-associated proteins DAXX to promote its degradation in human gliomas (Ryo et al., 2007). Pin1 induces cell death resistance function of BCL-2 and myeloid cell leukemia-1 (MCL-1) as anti-apoptosis factors (Basu and Haldar, 2002; Ding et al., 2008). Pin1 increases the survival of cisplatin-treated cervical cancer cells through Wnt/β-catenin and FoxM1 signaling (Wang et al., 2016). Pin1 increases the tamoxifen resistance upregulating LC-3 in breast cancer (Namgoong et al., 2010). Pin1 inhibit proapoptotic signals and activate antiapoptotic signals which consequently regulates the cell death resistance in cancer cells.

In allergen-injected rat, inhibition of Pin1 decreases the production of GM-CSF (Esnault et al., 2007). Pin1 induces the IL-22-induced proliferation and survival of breast cancer cells by activating c-Jun, and STAT3 (Kim et al., 2014). Pin1 is involved in inflammatory diseases such as non-alcoholic steatohepatitis (NASH) (Nakatsu et al., 2012), atherosclerosis (Paneni et al., 2015), rheumatoid arthritis (Jeong et al., 2009), and biliary cholangitis (Asuri et al., 2018).

## Therapeutic Targeting of PIN1

PIN1 is reported to be highly expressed in variety of human cancers, such as hepatic, prostate, lung, colorectal and esophageal cancers. It participates in diverse cancer-associated signaling pathways. Thus, the development of PIN1 inhibitors has been the focus of several research groups (Table 1).

**TABLE 1:** PIN1 inhibitors for cancer treatment.

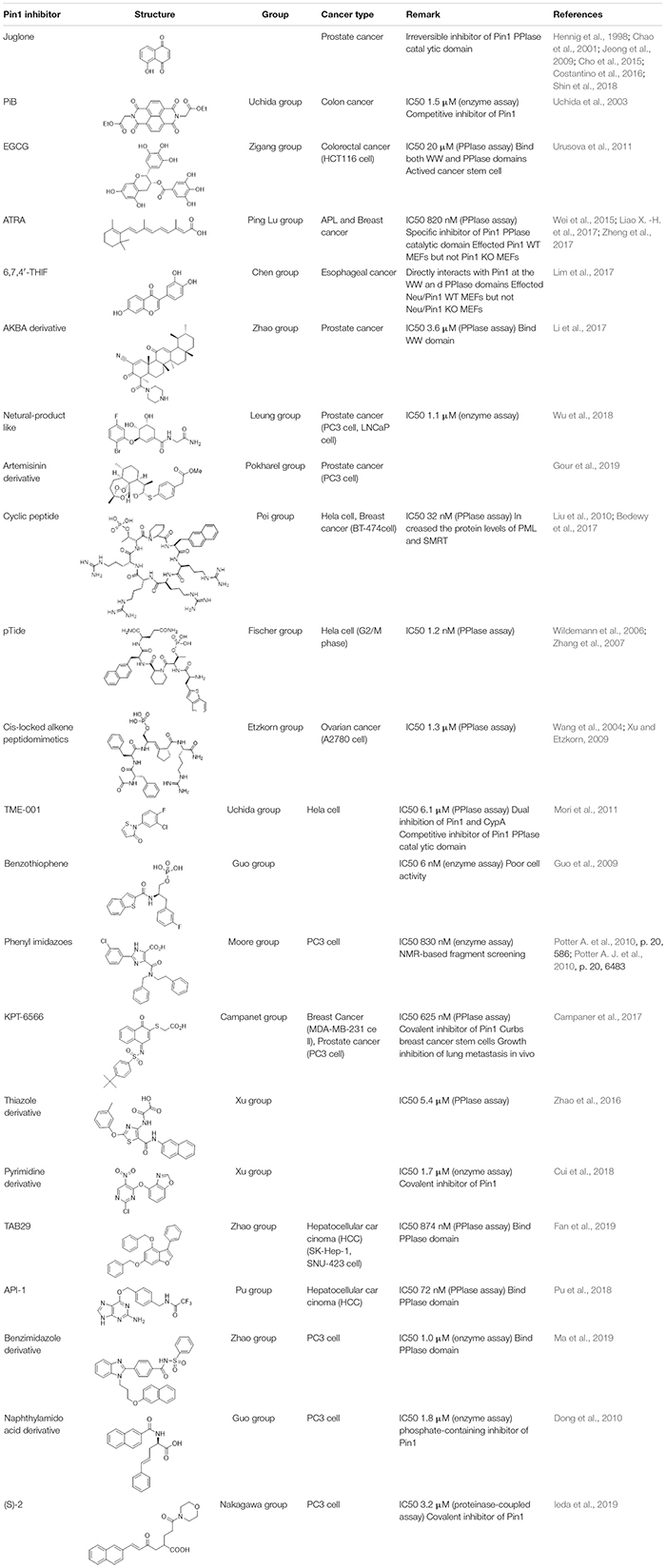

The first PIN1 inhibitor discovered by low-throughput screening is juglone. Juglone functions to inhibit the PIN1 PPIase activity in C-terminal catalytic domain, and a high dose of juglone reduces PIN1 protein expression. In addition, juglone has also shown to reduce the prostate cancer cell growth by inhibiting PIN1 activity (Hennig et al., 1998; Chao et al., 2001; Jeong et al., 2009; Costantino et al., 2016; Wang et al., 2017; Shin et al., 2018). Nevertheless, juglone possesses a primarily simple structure that may affect diverse specificity.

A chemically synthesized library containing compounds having a double-ring structure was screened and PiB inhibiting PIN1 (IC50 = 1.5 μM) was identified. Unlike juglone, PiB has been shown to a competitive inhibitor that inhibits the growth of *Pin1*-containing cells, but not that of *Pin1*-deficient cells. Furthermore, the inhibition of PIN1 by PiB treatment destabilizes Nanog, transcription factor required for the essential survival of cancer stem cells (Uchida et al., 2003). Uchida et al. identified TME-001 (IC50 = 6.1 μM) for a PIN1 inhibitor by library screening using *in vitro* enzymatic assay. The results revealed that this compound prevents the growth of HeLa cells (Mori et al., 2011).

Like juglone and PiB, other PIN1 inhibitors have been screened by low-throughput or high-throughput screening. pTide peptide shows PIN1 inhibition at 1.2 nM *in vitro*, but it is inactive in cells (Wildemann et al., 2006). The specificity of pTide against PIN1 has been shown by the X-ray crystal structure (Zhang et al., 2007). Attachment of an PIN1 octaarginine sequence to the pTide fragment enhances the membrane permeable ability and inhibits the cell growth in cancer (Liu et al., 2010).

A cyclic peptide derivative with increased cell permeable ability repressed the activity of PIN1 (IC50 = 32 nM) and inhibited the BT-474 breast cancer cell proliferation (Liu et al., 2010). Treatment of 100 nM of this peptide in cancer cell lines (HeLa and BT-474) increases the levels of PML and SMRT, and inhibits intracellular PIN1 activity (Liu et al., 2010; Bedewy et al., 2017). A major flavonoid of green tea, epigallocatechin 3 gallate (EGCG) is widely known as chemo-preventive compound for cancer and one of PIN1 inhibitor. Urusova et al. found utilizing X-ray crystal co-structure that EGCG binds to the N-terminal WW as well as C-terminal PPIase domains of PIN1. EGCG inhibits the activity of PIN1 *in vitro* enzyme assay (IC50 = 20 μM) and reduces JNK signaling pathway, and Bcl-xL and cyclin D1 expression in MEF cells transformed by ErbB2 (Urusova et al., 2011).

Structure-based design induces the identification of cis-locked alkene peptidomimetics as PIN1 inhibitors. They exhibited anti-proliferation activities in A2780 ovarian carcinoma cell line (Wang et al., 2004; Xu and Etzkorn, 2009). Using structure-based drug design, Vernalis and Pfizer develop small molecules. These inhibitors often contain a phosphate or carboxylate as isostere or a phenyl imidazole core, which is required to target the phosphate-binding pocket of the PIN1 protein (Guo et al., 2009; Dong et al., 2010; Potter A. et al., 2010; Potter A. J. et al., 2010; Guo et al., 2014). Pfizer has identified an inhibitor that repressed the PPIase activity of PIN1 at nano-molar concentrations (IC50 = 6 nM) by investigating and exploring the protein crystal structure of PIN1 (Guo et al., 2009; Dong et al., 2010; Guo et al., 2014). Using 900-number fragment library, Vernalis has developed a NMR-based fragment screen to isolate PIN1 inhibitors through the protease-coupled *in vitro* enzyme assay. A PIN1 inhibitor was synthesized (Potter A. J. et al., 2010) and showed good nanomolar inhibition against PIN1 *in vitro* (IC50 = 830 nM). However, they are poorly active or inactive in cell lines since the phosphate or carboxylate renders the inhibitors poor cell permeable ability (Guo et al., 2009; Dong et al., 2010; Potter A. et al., 2010; Potter A. J. et al., 2010).

Leung et al. have identified a PIN1 inhibitor from natural-product library using structure-based virtual screening and they show that compound 1 targets PIN1 and interferes the interaction of PIN1 with the NF-κB p65 subunit in cells. Moreover, a natural-product compound induced apoptosis in PC-3 cell lines (Wu et al., 2018). Using the virtual screening analysis, PIN1 protein has been identified as a target of 6,7,4′-trihydroxyisoflavone (6,7,4′-THIF). 6,7,4′-THIF bound to PIN1 protein, but did not bind to the family proteins such as FKBP or cyclophilin A, suggesting a selective and specific binding with PIN1. 6,7,4′-THIF compound was analyzed for specific inhibitory activity for PIN1 using *Neu/Pin1* knockout (KO) and *Neu/Pin1* wild-type (WT) MEFs. This PIN1 inhibitor affected *Neu/Pin1* WT MEF cells, but not *Neu/Pin1* KO MEF cells. In addition, the result of a xenograft tumor growth assay in mice utilizing *Neu/Pin1* KO and WT MEF cells have been shown similar to the result from the *in vitro* enzyme assay (Lim et al., 2017).

One of the reported inhibitors of PIN1, aetyl-11-keto-β-boswellic acid (AKBA) derivative has been shown to inhibit the growth of prostate cancer PC-3 (IC50 = 40 nM) and LNCaP (IC50 = 270 nM) cell lines. The compound inhibited the activity of PIN1, to stabilize cyclin D1, which improved anti-proliferative effects of prostate cancer treatment through new mechanisms (Li et al., 2017). In addition to the previous inhibitors of PIN1, thiazole derivatives (IC50 = 5.3 μM), pyrimidine derivatives (IC50 = 1.7 μM), benzimidazle derivatives (IC50 = 1.0 μM), 6-O-benzylguaninie derivative API-1 (IC 50 = 72 nM), and phenylbenzofuran derivative TAB29 (IC50 = 874 nM) have been reported as non-small molecule inhibitors (Zhao et al., 2016; Cui et al., 2018; Pu et al., 2018; Fan et al., 2019; Ma et al., 2019). In particular, *in silico* virtual screening was performed using the PIN1crystal structures and identified API-1 and TAB29 as small molecules that bind to the PPIase domain. Furthermore, PIN1 inhibition by API-1 and TAB29 upregulates miRNA biogenesis by maintaining the active XPO5 conformation and represses the development of hepatocellular carcinoma (HCC), suggesting that PIN1 mediates miRNA biogenesis mechanism, API-1 can be a drug candidate for therapy for *Pin1*-overexpressing or extracellular signal-regulated kinase-activated HCC (Pu et al., 2018; Fan et al., 2019).

The Food and Drug Administration (FDA) approved all-trans retinoic acid (ATRA) for acute promyelocytic leukemia (APL) therapy (Wei et al., 2015). ATRA was identified using a mechanism-based high-throughput screening system. ATRA inhibited the activity of PIN1 by binding with the C-terminal catalytic PPIase domain of PIN1. ATRA induces the degradation of PIN1 protein, but also suppresses the oncogenic function by decreasing the expression of cyclin D1. Furthermore, PIN1 inhibition mediated by ATRA induces the degradation of PML-RARA oncoprotein, resulting in anti-proliferative effect in APL cells and mouse models, as well as in humans. Moreover, a slow-release ATRA formulation induces the degradation of PIN1 and decreases tumorigenicity in mice xenograft model of HCC (Wei et al., 2015; Liao P. et al., 2017). Additionally, a combination of ATRA and sorafenib for the HCC treatment decreases the expression of PIN1 protein, increases cancer cell death, and represses the HCC growth compared with sorafenib or ATRA alone. These results provide an important rationale for further PIN1 inhibitor development to increase the therapeutic efficacy of general drug for HCC (Zheng et al., 2017).

A more recent study identifies KPT-6566, a novel PIN1 small molecule inhibitor, possessing high potency (IC50 = 625 nM) and specificity from a drug-like collection of 0.2 million commercial compounds (Campaner et al., 2017). Compounds capable of covalently binding to the C113 residue of the PIN1 catalytic domain were selected by virtual structure-based screening and cytotoxicity testing to select the final compounds. Structurally, the electrophile sulfonyl-acetate moiety of KPT-6566 directly faces the nucleophile sulfur atom of C113. Like ATRA, KPT-6566 also promotes the degradation of PIN1, resulting in the reduction of hyper-phosphorylated pRB and cyclin D1 levels. KPT-6566 increases the apoptosis and decreases the cancer cell proliferation such as pancreatic, lung, prostate, and breast cancers. It showed a better anti-proliferative effect on cancer cell lines than on normal cell lines. Furthermore, treatment with KPT-6566 inhibited the overexpression of *Pin1*, confirming the reduction of breast cancer stem cells. In addition, in *in vivo* studies, KPT-6566 has been shown to decrease the lung metastasis in breast cancer mouse models. Currently, KPT-6566 is the only PIN1 inhibitor in the preclinical stage of research.

A study reported by the Pokharel et al. shows that the artemisinin derivatives commonly used as antimalarial drugs are very effective in variety of cancer cell lines to inhibit cancer cell growth. Especially compound 9a, one of the artemisinin derivatives increases anti-proliferative, pro-apoptotic and anti-metastatic effect in PC-3 prostate cancer cells by decreasing the expression of Pin1, cyclin D1, c-Myc, elF4E, and PCNA (Gour et al., 2019).

Irreversible PIN1 inhibitor (*S*)-2 (IC50 = 3.2 μM), and its derivatives recently designed by Ieda et al. show the inhibition of Pin1 in protease-coupled *in vitro* assay and the reduction of cyclin D1 expression in PC-3 prostate cancer cell (Ieda et al., 2019).

## Conclusion

PIN1 is a well-known PPIase that regulates the cis–trans isomerization of pSer/Thr–Pro, which highlights its importance in the control of Pro-directed phosphorylation. PIN1 regulates protein function via conformational changes of target protein and is associated with the oncogenic pathway activation by controlling tumor suppressors and oncogenes. PIN1 is overexpressed in cancer tissues and CSCs, and correlated with poor clinical outcome in various cancer patients. Inhibition of PIN1 plays an important role in the tumorigenesis and angiogenesis of cancer, thereby providing a new great therapeutic target. Recently, PIN1 inhibitors have been developed elsewhere using structure-based drug designs and natural compounds that inhibit the activity of cancer. PIN1 obviously can be an super attractive target for curing cancer and cancer stem cells.

## Author Contributions

All authors designed and wrote this manuscript.

## Conflict of Interest

The authors declare that the research was conducted in the absence of any commercial or financial relationships that could be construed as a potential conflict of interest.
